# Comparison among Reconstruction Algorithms for Quantitative Analysis of ^11^C-Acetate Cardiac PET Imaging

**DOI:** 10.1155/2018/9193403

**Published:** 2018-02-28

**Authors:** Ximin Shi, Nan Li, Haiyan Ding, Yonghong Dang, Guilan Hu, Shuai Liu, Jie Cui, Yue Zhang, Fang Li, Hui Zhang, Li Huo

**Affiliations:** ^1^Department of Nuclear Medicine, Peking Union Medical College Hospital, Chinese Academy of Medical Science and Peking Union Medical College, 1 Shuaifuyuan, Dongcheng District, Beijing 100730, China; ^2^Sinounion Healthcare Inc., Building 3-B, Zhongguancun Dong Sheng International Pioneer Park, 1 Yongtaizhuang North Road, Haidian District, Beijing 100192, China; ^3^Center for Biomedical Imaging Research, Department of Biomedical Engineering, School of Medicine, Tsinghua University, Beijing 100084, China; ^4^Department of Biomedical Engineering, School of Medicine, Tsinghua University, Beijing 100084, China

## Abstract

**Objective:**

Kinetic modeling of dynamic ^11^C-acetate PET imaging provides quantitative information for myocardium assessment. The quality and quantitation of PET images are known to be dependent on PET reconstruction methods. This study aims to investigate the impacts of reconstruction algorithms on the quantitative analysis of dynamic ^11^C-acetate cardiac PET imaging.

**Methods:**

Suspected alcoholic cardiomyopathy patients (*N* = 24) underwent ^11^C-acetate dynamic PET imaging after low dose CT scan. PET images were reconstructed using four algorithms: filtered backprojection (FBP), ordered subsets expectation maximization (OSEM), OSEM with time-of-flight (TOF), and OSEM with both time-of-flight and point-spread-function (TPSF). Standardized uptake values (SUVs) at different time points were compared among images reconstructed using the four algorithms. Time-activity curves (TACs) in myocardium and blood pools of ventricles were generated from the dynamic image series. Kinetic parameters *K*_1_ and *k*_2_ were derived using a 1-tissue-compartment model for kinetic modeling of cardiac flow from ^11^C-acetate PET images.

**Results:**

Significant image quality improvement was found in the images reconstructed using iterative OSEM-type algorithms (OSME, TOF, and TPSF) compared with FBP. However, no statistical differences in SUVs were observed among the four reconstruction methods at the selected time points. Kinetic parameters *K*_1_ and *k*_2_ also exhibited no statistical difference among the four reconstruction algorithms in terms of mean value and standard deviation. However, for the correlation analysis, OSEM reconstruction presented relatively higher residual in correlation with FBP reconstruction compared with TOF and TPSF reconstruction, and TOF and TPSF reconstruction were highly correlated with each other.

**Conclusion:**

All the tested reconstruction algorithms performed similarly for quantitative analysis of ^11^C-acetate cardiac PET imaging. TOF and TPSF yielded highly consistent kinetic parameter results with superior image quality compared with FBP. OSEM was relatively less reliable. Both TOF and TPSF were recommended for cardiac ^11^C-acetate kinetic analysis.

## 1. Introduction

Dynamic PET imaging with kinetic analysis provides more quantitative information compared with the commonly used standardized uptake value (SUV) [1, 2]. Time-activity curves (TACs) in user-defined regions-of-interest (ROIs) can be extracted from a series of images and then applied onto the compartment kinetic models. Derived parameters reflect radiotracer exchange rate in the kinetic process and are considered as fully quantitative measurement of radiotracer metabolism [3, 4]. Fully quantitative analysis is currently limited in clinical research and drug development because of their complexity in terms of both experimental design and pharmacokinetic modeling methodology [5, 6].

Since TACs are generated from dynamic image series, the image quality is crucial to ensure the accurate and reliable quantitative analysis. The image quality is dependent on the choice of reconstruction algorithm. Classical analytical filtered backprojection algorithm (FBP) is straightforward but rarely used in clinic due to its poor image quality. Most current PET/CT systems employ fully 3D OSEM iterative reconstructions, which allow the corrections for random events, scatter events, normalization, dead time, and photon attenuations [7]. Utilizing more accurate physical system model, OSEM can greatly reduce the noise in the reconstruction and significantly improve the image quality [8, 9]. Recently, progress has been made on OSEM-type reconstruction by combining with time-of-flight (TOF) information [10, 11] and point-spread-function (PSF) kernel [12, 13] in the iterative reconstruction process. These new techniques substantially improve image quality and reduce the partial volume effect (PVE) [14].

Myocardial perfusion PET imaging provides quantitative information for myocardium assessment. Previous work has demonstrated that three-dimensional (3D) acquisition combined with attenuation-weighted ordered subsets expectation maximization (OSEM) reconstruction not only improves the image quality but also keeps the quantitative accuracy in cardiac ^18^F-FDG PET studies [15]. The quantitative influence of including TOF and PSF in OSEM algorithms as well as reconstruction parameters (subsets × iteration product; filters) for ^82^RB PET/CT perfusion studies is also investigated recently [16].

Among the variety of radiotracers, ^11^C-acetate is unique to measure the regional myocardial oxygen consumption and thus enables noninvasive quantification of the pathophysiology of cardiac metabolic alterations [17]. After bolus injection of ^11^C-acetate, myocytes extract ^11^C-acetate rapidly from the blood and then, via the tricarboxylic acid cycle in myocytes, the principal metabolite of acetate, ^11^CO2, is cleared from the myocardium. Accordingly, the TAC ascends rapidly and then declines. Using 1-tissue-compartment model, *K*_1_ and *k*_2_ can be calculated to represent the rate of uptake and washout of ^11^C-acetate. In addition, ^11^C-acetate has a physical half life of 20 min, and, combined with different frame lengths in the dynamic study, there is much difference in the count statistics in each frame in the acquired imaging data compared with ^18^F-FDG and ^82^RB PET studies, where different image reconstruction techniques may play a role in terms of quantitation. However, there are few studies that systematically investigate the quantitation performance of different reconstruction methods for cardiac ^11^C-acetate imaging.

The aim of this study was to explore the reconstruction-dependent image quality and their influences on kinetic analysis of ^11^C-acetate imaging. Image quality, semiquantitative analysis with SUV, and fully quantitative analysis with kinetic modeling parameters from four reconstruction algorithms were investigated: FBP, OSEM, OSEM method with time-of-flight information (TOF), and OSEM method modeled with both time-of-flight information and point-spread-function (TPSF).

## 2. Method

### 2.1. Patient and Scan Procedure

The study was approved by the Ethics Committee of Peking Union Medical College Hospital (PUMCH). Patients with suspected alcoholic cardiomyopathy (*N* = 24, male, age range, 48.3 ± 11.6 yrs) were enrolled in the study during January to June 2017. Written informed consent was obtained from all subjects.

All imaging was performed on a clinical PET-CT system [18] (PoleStar m660, Sinounion Healthcare Inc., Beijing, China) at PUMCH. ^11^C-acetate was synthesized as described in literature [19, 20]. After a low dose CT scan, a total of about 740 MBq ^11^C-acetate was administered intravenously to the subject and a 40 min dynamic PET scan was acquired for each patient.

### 2.2. Image Reconstruction

Each dynamic PET scan was sorted into 53 frames (15 × 10 s, 15 × 30 s, 16 × 60 s, and 7 × 120 s), and images were reconstructed with an object space of 192 × 192 × 117 and a voxel size of 3.15 × 3.15 × 1.87 mm^3^. Filtered backprojection (FBP) reconstruction and three iterative reconstruction methods, that is, OSEM, OSEM with time-of-flight, and OSEM with both time-of-flight and point-spread-function, were performed for each study. The four reconstruction methods were named as FBP, OSEM, TOF, and TPSF, respectively, for the comparison in this study. Necessary corrections such as random, attenuation, scatter, normalization, dead time, and decay corrections were applied. For the FBP method, scatters and randoms were directly subtracted from projections while, for the iterative methods, they were included in the iteration. The iterative equation used in the ordinary Poisson OSEM-type algorithms was [7]

(1) $$\begin{array}{c} f_{j}^{\left(k,q+1\right)}=\frac{f_{j}^{\left(k,q\right)}}{\sum_{i\in S_{q}}A_{ij}}\overset{}{\underset{i\in S_{q}}{\sum }}\frac{A_{ij}p_{i}}{\sum_{l=1}^{M}A_{il}f_{l}^{\left(k,q\right)}+r_{i}+s_{i}}, \end{array}$$

where *f* = [*f*_1_, *f*_2_,…,*f*_*M*_]^*T*^ was the estimated tracer intensities in a finite discrete object space. The sinogram data, *p* = [*p*_1_, *p*_2_,…,*p*_*N*_]^*T*^, was a collection of detected coincidence events with independent Poisson statistical distributions. In (1),* k* is the iteration number and *q* is the subset index. The whole sinogram data was divided into several subsets, denoted as {*S*_*q*_}, in order to increase the convergence speed. In general, subsets are evenly distributed with angular bins. In the case of both TOF and TPSF, time-of-flight information was treated as an additional dimension of time-of-flight bin in the sinogram space. *r* and *s* represented expectations of additive random and scatter events. *A* was the discrete system response function defined by the physical characteristics of imaging system, which in practice was factorized as a product of normalized correction matrix, attenuation correction matrix, and geometrical modeling matrix. For the TPSF, an additional product of the point-spread-function matrix was added to simulate the depth of interaction in crystals [21, 22].

In iterative reconstructions, the image quality was controlled by the combination of subsets and iterations, which can be simplified as expectation maximization equivalent (EM-equivalent) iterations (the product of iterations and subsets) [23]. More EM-equivalent iterations result in less blurred and noisier reconstructed images. The optimization of EM-equivalent iterations depends on a variety of variables, such as activity distribution, the use of time-of-flight information, point-spread-function modeling, and noise equivalent counts in data.

To better analyze the effects of EM-equivalent iterations on the characteristics of the reconstructed images, we estimated the noise level in the myocardium and the contrast obtained between myocardium and blood pool in LV. One patient was randomly selected and a 30 s PET acquisition (the 21st frame) was used for the evaluation. For simplicity, the subsets were fixed to be 10 in the study. Mean myocardial counts (signal mean) and related standard deviations (signal SD) were calculated from the myocardial ROIs. Background mean counts (background mean) were defined as the average from the blood-pool ROI in LV. The definitions of ROIs were detailed in the next section. The contrast and noise properties in three iterative reconstructions were evaluated as a function of the number of iterations. The coefficient of variance (CV) and contrast were calculated as follows [23]:

(2) CV=signal  SDsignal  meancontrast=signal  mean−background  meanbackground  mean.

Furthermore, the convergence performances of the fitted kinetic parameters for the three iterative reconstruction methods were explored. In the analysis, the entire image frames were used to minimize the effect of noise in the reconstructed images. In the study, the same patient study data as mentioned above was used and the subsets in the reconstructions were fixed to be 10 as well.

Based on the convergence behaviors with respect to image quality and kinetic parameters, the optimum iterations were determined, respectively, for the three iterative reconstruction methods. The post-filter also affects image quality and the FWHM of post-filter were adjusted separately for different reconstruction methods to obtain similar image quality.

### 2.3. Image Analysis, Kinetic Modeling, and Statistics

**Figure 1:**
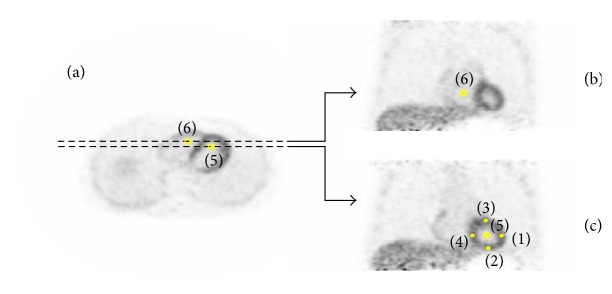
PET images reconstructed by TOF protocol: a transversal slice (a), a coronal slice along the higher dashed line (b), a coronal slice along the lower dashed line (c). The iterative reconstruction was performed with 10 iterations and 10 subsets, and a 5 mm (FWHM) Gaussian filter was applied (axial and transaxial) after reconstruction. The scan started 450 seconds after ^11^C-acetate injection with the scan duration of 30 seconds. The four myocardial ROIs with 5 mm in diameter were evenly distributed along the myocardial wall (ROI (1)–(4)) and the two blood-pool ROIs with 10 mm in diameter were located in the center of the left ventricle (ROI (5)) and right ventricle (ROI (6)).

Spherical regions-of-interest (ROIs) were manually drawn in the reconstructed images by experienced clinicians. As shown in Figure 1, four spherical ROIs (indexes (1)–(4)) with a diameter of 5 mm were drawn to calculate myocardium uptake and they were evenly distributed around LV myocardium and close to epicardium to avoid possible contamination from the blood pool. It is worthwhile to note that, for myocardial ROI analysis in this study, statistics were obtained by averaging the four 5 mm spherical ROIs to reduce the influences of partial volume effect (PVE), statistical noise, and potential motion artifacts.

In order to explore the influence of different reconstruction methods on the quantitation of dynamic cardiac PET images, SUVs of the myocardial ROI at different times after tracer injection were first analyzed. For each reconstruction, four discrete time points were selected representing the high (2 min), moderate (5 min and 10 min), and low (30 min) myocardium activity concentrations, respectively. As explained earlier in the text, mean activity concentration for each time point was obtained by averaging the four 5 mm myocardial ROIs. The corresponding SUV was defined as SUV_mean_ in later comparison and analysis.

**Figure 2:**
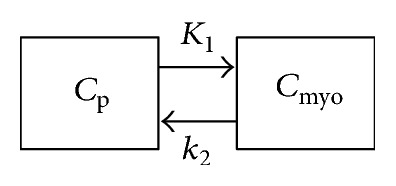
The 1-tissue-compartment model for kinetic modeling of ^11^C-acetate cardiac flow from PET images. *C*_*P*_ and *C*_myo_ represent the tracer concentration in plasma and myocardial tissue, respectively. *K*_1_ is proportional to perfusion value and *k*_2_ is related to oxidative metabolism.

Kinetic analysis was also performed on the dynamic image series with different reconstruction methods. Kinetic analysis requires an accurate input function to estimate tracer activity concentration of blood. The gold standard is to collect blood samples by arterial cannulation in order to have direct activity concentration measurement. However, this approach induces patient discomfort as well as complexity during scan. In the study, a 1-tissue-compartment model [24], as shown in Figure 2, was used for kinetic modeling of ^11^C-acetate cardiac flow from PET images as a feasible and noninvasive alterative to arterial cannulation. A spherical ROI with a diameter of 10 mm (index (5) in Figure 1) was manually drawn inside the left ventricle (LV) blood pool to calculate the tracer concentration in plasma. For each reconstruction method, corresponding blood and myocardial time-activity curves (TACs) were extracted from the dynamic image series as the image-derived input functions for kinetic analysis. Also to note is that the myocardial TAC was obtained by averaging the four 5 mm myocardial ROIs as explained earlier.

Kinetic modeling was performed on TACs in LV blood-pool ROI and averaged myocardial ROI using the PMOD software (PMOD Technologies, Zurich, Switzerland). The 1-tissue-compartment model for ^11^C-acetate cardiac flow implemented in PMOD was adopted to calculate the *K*_1_ and *k*_2_ parameters of ^11^C-acetate, where *K*_1_ is proportional to perfusion value and *k*_2_ is related to oxidative metabolism. For the purpose of spillover correction, a 10 mm diameter spherical ROI (index (6) in Figure 1) was manually drawn inside the right ventricle (RV) blood pool. This RV blood-pool ROI and LV blood-pool ROI were used by the PMOD software for automatic correction of the spillover from LV and RV into the myocardium [25].

Correlation relationships among kinetic parameters were investigated using linear regression. Group mean (*μ*) and standard deviation (*σ*) of kinetic parameters and SUVs for all subjects were compared among different reconstructions. Statistical analysis was performed using paired, two-sample Student's *t*-test (or Welch's *t*-test) [26] with a significance level *α* of 0.05.

## 3. Results

### 3.1. Iterations Optimization

**Figure 3:**
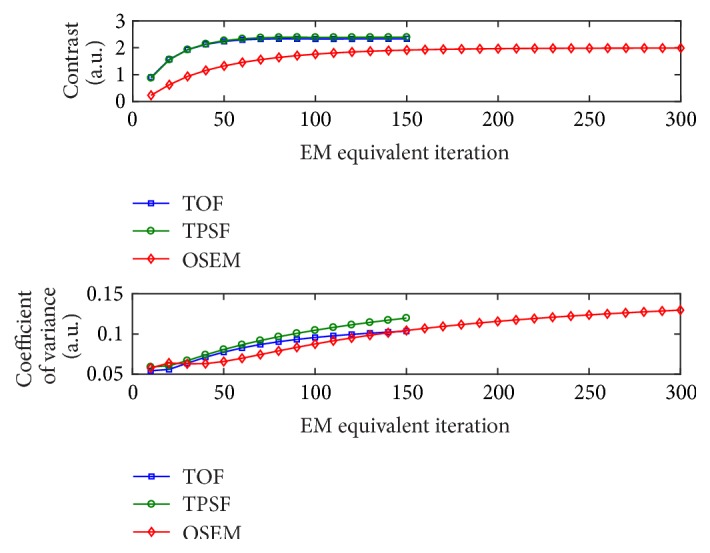
The contrast and noise level measured by the coefficient of variance as a function of EM-equivalent iterations for iterative reconstructions.

Figure 3 showed the convergence behaviors of image noise and contrast for the three iterative algorithms plotted against EM-equivalent iteration. Note that all the iterative reconstructions in this study were performed with 10 subsets. Compared with OSEM, TOF increased speed of reconstruction algorithm convergence, which is in accordance with previous study in [10]. The incorporation of PSF modeling into reconstruction affected the convergence speed of image noise more than contrast. In this study, to ensure good contrast convergence and then the minimum noise property, the number of EM-equivalent iterations of 200 (corresponding to the iteration number of 20) was chosen for the OSEM method while the number of EM-equivalent iterations of 100 (corresponding to the iteration number of 10) was chosen for the TOF and TPSF methods. Furthermore, in order to minimize the difference in CV and obtain similar image qualities, a 3D Gaussian filter of 5.0 mm in FWHM was applied to both TOF and TPSF images, while a 3D Gaussian filter of 6.5 mm in FWHM was applied to OSEM images.

Visual qualitative assessments on the reconstructed images were performed by experienced clinicians using double blinded study. The contour of LV wall, ^11^C-acetate distribution in ventricular myocardium, and streak artifacts in cardiac region, especially in LV cavity, were carefully examined.

**Figure 4:**
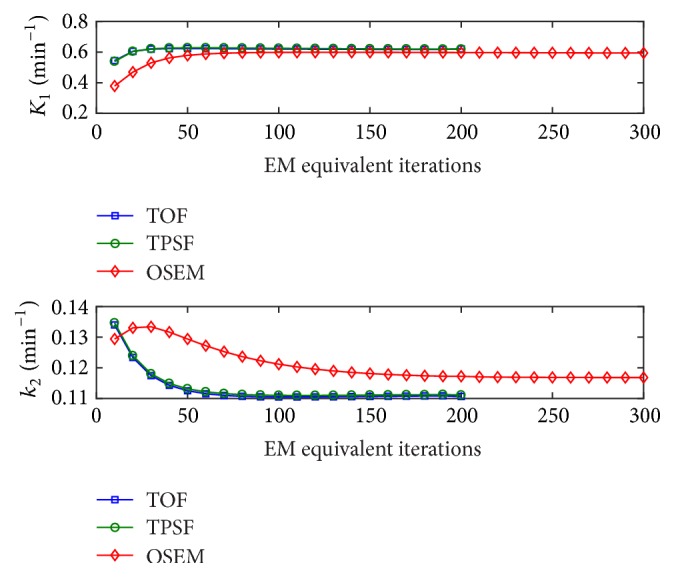
The kinetic parameters (*K*_1_ and *k*_2_) as a function of EM-equivalent iterations for iterative reconstructions.

The determinations of EM-equivalent iteration were further verified in the convergence analysis of kinetic parameters.  Figure 4 displayed the convergence behaviors of kinetic parameters (*K*_1_ and *k*_2_) as a function of EM-equivalent iterations for the three iterative reconstruction algorithms. As shown in the figure, the EM-equivalent iterations of 100 ensured the convergence of the kinetic parameters for both TOF and TPSF cases. Although *K*_1_ and *k*_2_ for OSEM converged slowly, the number of EM-equivalent iterations of 200 was still large enough to obtain the stable values.

### 3.2. Reconstruction Images

**Figure 5:**
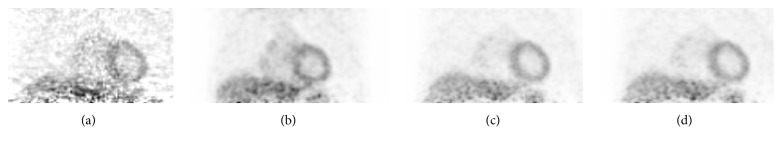
Coronal PET images reconstructed by FBP (a), OSEM (b), TOF (c), and TPSF (d). OSEM reconstruction was performed with 20 iterations and 10 subsets, with application of a 6.5 mm (FWHM) Gaussian post-filter. Both TOF and TPSF reconstructions were performed with 10 iterations and 10 subsets, with application of a 5 mm (FWHM) Gaussian post-filter. The scan started at 450 seconds after ^11^C-acetate injection with scan duration of 30 seconds.

Figure 5 showed the representative coronal PET images reconstructed using FBP, OSEM, TOF, and TPSF, respectively. The image frame started at 450 seconds after ^11^C-acetate injection with a duration of 30 seconds. FBP method exhibited the poorest image quality with streak artifacts, especially in low count regions. As a comparison, iterative OSEM-type algorithms (OSEM, TOF, and TPSF) showed significant improvements in terms of image quality. Residual activity in the LV was obvious in the OSEM method, and the LV myocardium uptake exhibited much nonhomogeneity. The incorporation of time-of-flight information in the reconstruction could greatly reduce the activity bias and led to a more continuous contour of ventricles and homogeneous myocardium uptake, as demonstrated in the TOF and TPSF images. Furthermore, time-of-flight reconstruction leads to improved convergence in cold region, resulting in much more clear blood pools in the LV in the TOF and TPSF images. Similar image qualities were observed between TOF and TPSF methods, suggesting that the incorporation of PSF does not contribute significantly to cardiac PET images.

### 3.3. SUV and Kinetic Analysis

**Figure 6:**
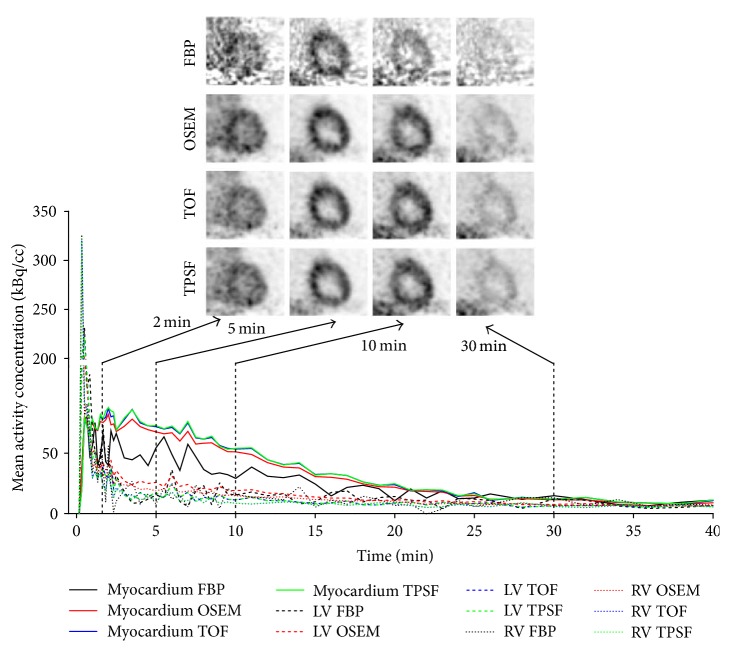
Representative heart images reconstructed from four methods at different time points, as well as the corresponding TACs from myocardium (in solid lines), the LV (in dashed lines), and the RV (in dotted lines) after the injection of ^11^C-acetate.

**Figure 7:**
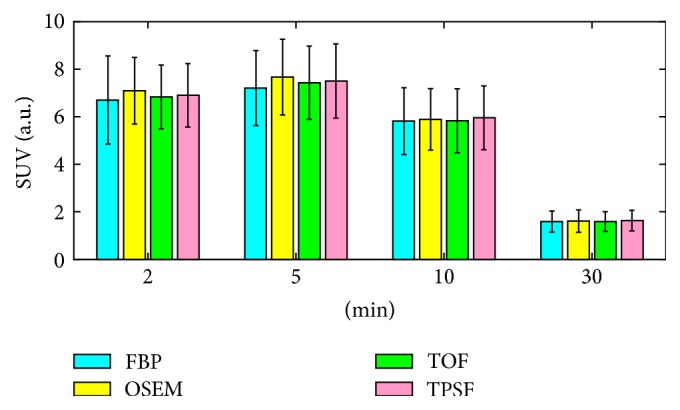
Means and standard deviations of semiquantitative ROI-based SUV from four reconstruction methods at 2 min, 5 min, 10 min, and 30 min after radiotracer injection.

Figure 6 showed the magnified reconstructed images with different reconstruction methods at 2 min, 5 min, 10 min, and 30 min after tracer injection. For each reconstruction method, the group mean and standard variation (SD) of SUV_mean_ from all the patients were shown in Figure 7. SD with the FBP method was the largest while the SDs with the three OSEM-type methods were similar. The OSEM method exhibited the largest bias on the mean value. Overall, similar performances were observed in the TOF and TPSF methods. At 30 min after injection, with a scan duration of 120 s, the four methods displayed almost the same SUV_mean_ due to the excellent SNR in measurement.

Figure 6 also showed the TACs of the myocardium tissue and the blood pools in the LV and RV extracted with the four reconstruction methods. The FBP method generated much oscillation in the TACs especially in the early frames due to its intrinsic sensitivity to statistical noise. The nonnegative constraints in iterative OSEM-type methods led to positive biased estimation on the regional activity concentration in low count PET data, while the FBP method yields both positive and negative values. Thus the TACs of the iterative methods always lied on the top of the TAC of the FBP method. The incorporation of time-of-flight information in the TOF and TPSF methods could greatly reduce the influence of statistical noise, resulting in much less divergent TACs compared with the OSEM method. The TACs of the TOF and TPSF methods were very similar and, again, the benefit of incorporating PSF was not obvious.

**Figure 8:**
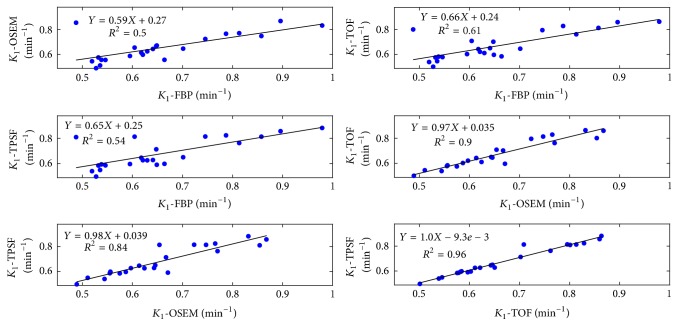
Linear regressions of myocardium *K*_1_ calculated from reconstruction methods. Each point represented the corresponding kinetic parameter in myocardium ROI of one patient. Linear function with slope rate, intercept, and squares of Pearson correlation coefficients were indicated.

**Figure 9:**
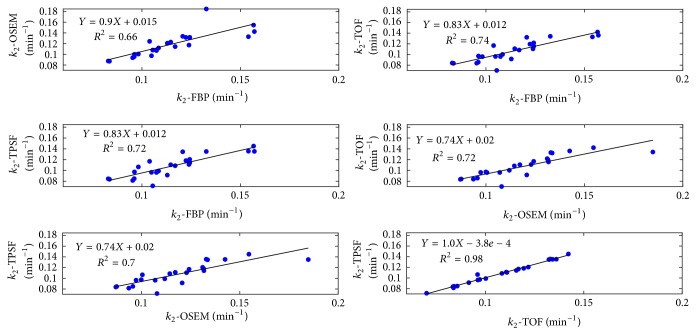
Linear regressions of myocardium *k*_2_ calculated from reconstruction methods. Each point represented the corresponding kinetic parameter in myocardium ROI of one patient. Linear function with slope rate, intercept, and squares of Pearson correlation coefficients were indicated.

Figures 8 and 9 illustrated the linear regressions of all the kinetic parameters *K*_1_ and *k*_2_, using the TACs shown in Figure 6. Overall, kinetic parameters derived from FBP reconstruction were the least relevant to those from the OSEM-type reconstructions. This was because FBP approach produced divergent TACs due to the statistical noise and thus introduced extra variations in curve fitting and kinetic modeling. Both TOF and TPSF methods had excellent linear correlations for all kinetic parameters, and they had slightly less correlations with OSEM method.

**Figure 10:**
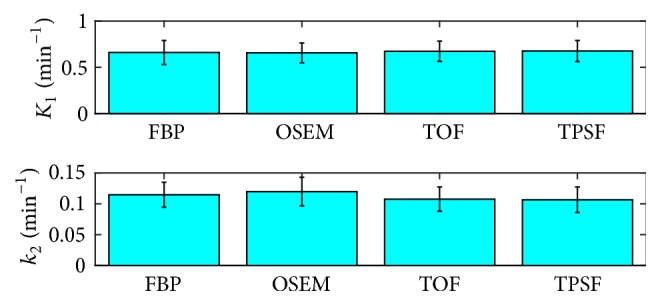
Means and standard deviations of myocardiums *K*_1_ and *k*_2_ calculated using image-derived input functions for 24 patients with respect to four reconstruction methods of FBP, OSEM, TOF, and TPSF.

Group comparison of the mean values and standard deviations of myocardiums *K*_1_ and *k*_2_ were shown in Figure 10. Consistent kinetic parameters were obtained from the TOF and TPSF methods. The kinetic parameters derived from the FBP method had comparable mean values but relatively the largest SD for *K*_1_.

The statistic analysis for kinetic parameters was summarized in Table 1. No significant difference was found among different reconstruction approaches. In terms of parameters *K*_1_ and *k*_2_, the OSEM method presented relatively higher residual in correlation with the FBP method compared with the TOF and TPSF methods. The TOF and TPSF methods were highly correlated with each other with the largest *P* values. No significant difference was found among all the reconstruction methods in the SUV_mean_ test (Table 2). The results implied that all the reconstruction methods reported similar SUVs in the PET images.

## 4. Discussion

In the presented work, ^11^C-acetate cardiac dynamic PET imaging studies were carried out. Data were acquired and reconstructed offline with FBP, OSEM, TOF, and TPSF methods. Both SUVs and kinetic parameters were investigated to evaluate the impacts of different reconstruction approach. It is well known that iterative reconstruction leads to significant improvement in image quality compared with FBP reconstruction, as demonstrated in Figures 5 and 6. However, FBP is much faster in calculation and may be attractive in dynamic studies where iterative methods usually take hours to reconstruct the dynamic image series. Moreover, FBP is linear and does not produce noise-induced positive bias. Despite its poor image quality and visual artifacts, FBP images can be quantitatively accurate in mean values when the ROI is chosen to be large enough to be less affected by PVE, that is, spill-in and spill-out effects due to variable intense activity surrounding tissues [6]. In this study, all the voxels in the myocardial ROIs were averaged to reduce the noise effect and thus counteract the contributions of positive and negative pulses on closer neighboring streaks. In low count PET images, the SUVs were diverged by the noise, but the group average of SUV_mean_ could be considered accurate due to the linear noise property and the permit of negative values. In comparison, OSEM-type methods had some remaining positive bias in case of very noisy data due to the inherent nonnegativity constraint. The group comparison on SUV among different reconstructions showed that FBP reconstruction had the largest standard variation. TOF reconstruction behaved more similar to FBP reconstruction for all the time point results in terms of mean value of SUV, especially at early frames, which implies that TOF reconstruction is insensitive to the statistic noise. No statistical significance was found from all the time point SUVs of all the reconstructions. TOF reconstruction was better than the other iterative method such as OSEM or TPSF.

SUV_mean_ was chosen in this study instead of SUV_peak_ or SUV_max_ [27]. Using mean values in ROIs reduces the dependency on the statistical quality of the images, but the averaging process also causes loss of sensitivity to hot spot. For clinical examination, the peak values are normally used where hot spots usually attract more attention. However, for kinetic analysis, mean values appear to be more appropriate in order to be less sensitive to statistical noise normally seen in the dynamic acquisition.

In this study, the influence of different reconstruction methods on the kinetic analysis was also investigated. It is known that the noise level in the TAC will affect the robustness of the kinetic parameter estimation. As shown in Figure 6, the TACs from FBP reconstruction exhibited much oscillation, especially in the early frames, leading to the largest standard variation and the worst correlation in terms of kinetic parameters compared with other reconstruction methods.

Compared with FBP, TACs from iterative methods were much smoother. For the kinetic parameter *k*_2_, differences were seen in the OSEM method compared with both TOF and TPSF methods. OSEM reconstruction has been widely accepted by clinics and has been incorporated in most commercial PET/CT scanners. However, in this study, we found that OSEM method performed poorly in the quantitative analysis of cardiac imaging. One possible reason is that the blood pools in the OSEM reconstructed images exhibit residual activities, which affects the blood input function for the kinetic modeling. The incorporation of time-of-flight information could reduce the noise and lead to much less divergence in the TACs compared with the OSEM method. With time-of-flight information, the positions of annihilation along lines of response can be approximately determined. With this additional information, the reconstructed PET images could achieve better image quality and low count recovery, thus improving the accuracy of regional activity quantification.

In this study, the advantage of incorporating point-spread-function in the reconstruction was not obvious in the quantitative analysis. The point-spread-function describes the detector response of incident photon. The implementation of point-spread-function kernel in the reconstruction often helps to improve the spatial resolution and reduce the partial volume effect. And usually it helps to delineate boundaries with sharp intensity transitions. However, in this study, the defined ROIs were surrounded by almost uniform activity in myocardium and there were no sharp activity transitions along the ROI boundaries. In addition, the partial volume effect had been largely reduced since the size of ROIs was approximately twice the spatial resolution of PET scanner. As a result, similar TACs and good correlations in all the kinetic parameters were found between the TPSF and TOF results. And the two methods generated consistent mean values for all the kinetic parameters with small standard deviations. Similar results were also seen in terms of image quality between the two reconstruction methods.

In this study, neither cardiac nor respiratory gating was used during data acquisition due to the high counts statistical variance especially in early frames, which will become worse as the data are further divided into phases for the gating process. However, the possible unconscious body motion still causes concern for the 40 min long dynamic data acquisition. It is worthwhile to note that, in the ROI analysis in this study, ROIs were manually defined on a single frame and applied to all the image series for each patient. It was possible that the myocardium ROIs partially entered the blood pool in some image series due to patient's unconscious motion. For simplicity these motions were not corrected frame by frame in the study. In order to evaluate the motion effect, data of one patient with obvious movement was examined and two myocardium TACs were generated using fixed ROI positions across image frames as well as manually adjusting ROI positions when necessary to move myocardium ROIs away from the blood pool. The two sets of kinetic parameters derived from the two TACs were compared and results were shown in Table 3. No significant differences were observed for iterative reconstruction methods, while, for the FBP method, the difference was more obvious. The possible reason may be that kinetic parameters derived from the divergent TAC from the FBP method are more sensitive to deviations in the curve. However, since patient's unconscious motion was not evident in most cases, ROI with fixed positions was used in the evaluation for all reconstruction methods including FBP in the study.

## 5. Conclusion

All the tested PET reconstruction algorithms performed similarly in the SUV analysis at the selected time points. Kinetic parameters *K*_1_ and *k*_2_ also exhibited no statistical difference among the four reconstruction algorithms in terms of mean value and standard deviation. However, for the correlation analysis, OSEM reconstruction presented relative higher residual in correlation with FBP reconstruction compared with TOF and TPSF reconstruction. TOF and TPSF reconstruction were highly correlated with each other and led to highly consistent kinetic results with superior image quality. As a result, both TOF and TPSF are suitable for cardiac ^11^C-acetate kinetic analysis. This conclusion needs to be further validated in the future study when arterial blood sampling is provided to measure the true time-activity curve as input function for kinetic modeling.

## Figures and Tables

**Table 1:** Paired two-sample Welch's *T*-test Results for kinetic parameters with respect to combinations of every two reconstruction approaches.

	FBP-OSEM	FBP-TOF	FBP-TPSF	OSEM-TOF	OSEM-TPSF	TOF-TPSF
*K*_1_	0.92	0.74	0.59	0.63	0.48	0.81
*k*_2_	0.55	0.21	0.25	0.08	0.10	0.93

**Table 2:** Paired two-sample Welch's *T*-test results for SUV_mean_ at four time points with respect to combinations of every two reconstruction approaches.

	FBP-OSEM	FBP-TOF	FBP-TPSF	OSEM-TOF	OSEM-TPSF	TOF-TPSF
2 min	0.40	0.74	0.64	0.53	0.64	0.87
5 min	0.34	0.66	0.55	0.59	0.71	0.87
10 min	0.86	0.98	0.73	0.87	0.86	0.85
30 min	0.91	0.94	0.80	0.85	0.90	0.94

**Table 3:** Comparisons of kinetic parameters with and without ROI movements by four reconstruction approaches^1^.

	FBP	OSEM	TOF	TPSF
*K*_1_	**0.71**	**0.85**	**0.80**	**0.81**
	*0.59*	*0.86*	*0.79*	*0.80*
*k*_2_	**0.13**	**0.18**	**0.13**	**0.14**
	*0.10*	*0.17*	*0.14*	*0.14*

^1^The parameters with/without ROI movements are shown in bold/italic tables.
